# The Teledentistry, Impact, Current Trends, and Application in Dentistry: A Global Study

**DOI:** 10.1155/2021/5437237

**Published:** 2021-10-25

**Authors:** Afsheen Maqsood, Muhammad Shahrukh Khan Sadiq, Daud Mirza, Naseer Ahmed, Abhishek Lal, Mohammad Khursheed Alam, Mohamad Syahrizal Bin Halim

**Affiliations:** ^1^Department of Oral Pathology, Bahria University Medical and Dental College, Karachi 75530, Pakistan; ^2^Prosthodontics Unit, School of Dental Sciences, Health Campus, Universiti Sains Malaysia, 16150 Kubang Kerian, Kota Bharu, Kelantan, Malaysia; ^3^Department of Prosthodontics, Altamash Institute of Dental Medicine, Karachi 75500, Pakistan; ^4^Research Intern, Department of Prosthodontics, Altamash Institute of Dental Medicine, Karachi 75500, Pakistan; ^5^Department of Preventive Dentistry, College of Dentistry, Jouf University, Sakaka, Al Jouf, 72345, Saudi Arabia; ^6^Conservative Dentistry Unit, School of Dental Sciences, Health Campus, Universiti Sains Malaysia, 16150 Kubang Kerian, Kota Bharu, Kelantan, Malaysia

## Abstract

**Objective:**

The present study was aimed at assessing the impact of teledentistry, its application, and trends in uplifting dental practice and clinical care around the world. *Material and Methods*. The present observational study comprised of an electronic survey distributed among dental professionals around the globe. The validated survey form consisted of a total 26 questions with 5-point Likert scale response. The questionnaire used was divided into four domains: usefulness of teledentistry for patients, its usefulness in dental practice, its capacity to improve the existing practice, and the concerns attached to its use. The statistical analysis was performed using SPSS-25. ANOVA test was used to assess the effect of independent variables on dependent variables. A *p* value of ≤0.05 was taken as statistically significant.

**Results:**

A total of 506 dental professionals participated in the study with the response rate of 89.39%. More than half of the participants (50-75%) endorsed that teledentistry is a useful tool for improving clinical practice as well as patient care. Two-thirds of the participants (69.96%) considered that teledentistry would reduce cost for the dental practices. On the other hand, about 50-70% of dental professionals expressed their concerns regarding the security of the data and consent of patients. The most preferred communication tool for teledentistry was reported to be videoconference followed by phone. The majority of participants recommended the use of teledentistry in the specialty of oral medicine, operative dentistry, and periodontics. There was a significant difference between the age, experience of dentists, and their qualifications with domains of teledentistry.

**Conclusions:**

The overall impact of dental professionals towards teledentistry was positive with adequate willingness to incorporate this modality in their clinical practice. However, the perceived concerns pertaining to teledentistry are significant impediments towards its integration within the oral health system. An in-depth study of its business model and cost-benefit needs of time, especially in the context of developing countries, in order to avail the optimum benefits of teledentistry.

## 1. Introduction

Since the advent of telehealth technology decades ago, the fields of medicine and dentistry have seen substantial scientific advances. The use of telehealth-associated modalities has transfigured diagnosis, therapies, and surgery in the field of dentistry [1]. In that regard, teledentistry (TD) is a form of telehealth utilizing a combination of telecommunications and dentistry, which involves the exchange of clinical information and relevant imaging over remote distances for consultation and treatment planning. Teledentistry is a novel field with a massive potential for uplifting clinical care and dental education with its innovative approach [2].

The first practical application of TD has its root in a US project launched in 1994 to assess the dental health of the servicemen of United States army [3]. The term TD was formally used for the first time in the literature by Cook in 1997. The initial description pertaining to TD was confined to videoconference and its associated role in consultation and diagnosis from long distance [4]. With rapidly improving technology in the 21^st^ century, the term TD expanded its ambit to include the subdomains such telediagnosis teleconsultation, teletriage, and telemonitoring [5]. According to the American Dental Association, TD comprises of four basic modalities that include synchronous, asynchronous, remote patient monitoring, and mobile health. The synchronous modality utilizes virtual video call in order to facilitate real-time interaction between the dental practitioner and patient, while the asynchronous approach deals with diagnosis and examination through the transfer of data via videos, radiographs, and intraoral imaging [6].

The COVID-19 pandemic caused due to the spread of SARS-COV-2 virus has posed a menacing challenge to the healthcare systems across the world. Due to its transmission via droplets and air, the traditional face-to-face interaction between the dental practitioners and patient entails a risk of viral transmission [7]. In such circumstances, TD has proven itself to be a boon; it has circumvented the traditional face-to-face dentist-patient interaction by providing an effective substitute for the purpose of online consultation, exchange of investigations, and planning treatment [8]. Ample evidence has universally accepted TD as a viable modality that provides minimal cost, reduced stress of transportation, and better access to specialist practice [9].

Numerous studies have been conducted in various countries regarding the perceptions, effectiveness, and applications of TD at national level. Estai et al. assessed the perception of Australian dentists on the use of TD and concluded that an overwhelming majority of dentists (80%) agreed regarding the beneficial outcomes of using TD for both dentists and patients [10]. A Canadian study conducted by Palmer et al. showed similar results where the majority of orthodontists supported the use of digital and electronic technology in dental practice [11]. A recent questionnaire-based study carried out in Saudi Arabia reported that a substantial proportion of respondents agreed with the fact that TD would improve dental practice through enhancing communication with peers, guidance, and referral of new patients despite the concerns of data privacy and security [12].

So far, the current literature is devoid of a global survey that is focused upon assessing the usefulness of TD in provision of dental care. A study incorporating the impact of TD and current trends among dental practitioners at global stage needs to time in order to evaluate the application and effectiveness of TD in different countries and their respective dental healthcare systems. Therefore, the current study was aimed at evaluating the impact of TD, its application, and trends in improving dental practice and patient outcomes.

## 2. Materials and Methods

### 2.1. Study Setting and Ethical Consideration

The present study was approved by the ethical review committee of Altamash Institute of Dental Medicine, Karachi. The study was carried out at numerous countries.

### 2.2. Sample Size and Study Design

The sample size was calculated through OpenEpi software. Consider the usefulness of teledentistry for patients, with a mean score value of9.64 ± 4.26[12] and with a 95% confidence interval and the power of test 80%. The total estimated sample size was 506 participants. The study design was descriptive observational that comprised of convenient sample of 506 dental professionals.

### 2.3. Questionnaire Design and Distribution

An electronic and validated questionnaire was disseminated among the selected dental professionals between June and July of 2021 through e-mail and other social media applications (WhatsApp®, Facebook®, Instagram®, skype®, Imo messenger®, snapchat®, and LinkedIn®). The permission from dentists to participate in the study was sought out before the questionnaire dissemination. The dentists working in dental hospitals, clinics, and institutes were approached through phone calls and emails for this purpose. The questionnaire used in this study was adopted after a prior permission, from a similar study conducted in the Kingdom of Saudi Arabia by Al Khalifa and AlSheikh [12]. The questionnaire consisted of two parts related to participant's general information and different domains of teledentistry. The first part of the questionnaire covered professional, demographic information, and communication method preferences. The second part of the questionnaire was based on five-point Likert-type responses. This part was comprised of a total of 26 questions, which were further divided under four domains that encompassed: data security concerns by the dental professionals, teledentistry and practice improvement, the usefulness of teledentistry for dental practice, and its usefulness for dental patients.

There was a brief description of the questionnaire's purpose with a definition of teledentistry and its benefits and possible uses in daily practice. The consent agreement was incorporated within the questionnaire. The regular reminders (after an interval of week) were sent to the nonrespondents via e-mail and other social media networks, after initial distribution of the questionnaire. In response, 566 forms were received back from the participants, out of which 60 incomplete forms were excluded from the study. A total of 506 forms were included in the study.

### 2.4. Statistical Analysis

The SPSS-25 was used for statistical analysis. Descriptive statistics were performed for frequency, percentage, mean, and standard deviation of demographic variables; qualification of participants; experience; place of practice; and use of teledentistry in different specialties. ANOVA test was applied to see the effect of independent variables (age, gender, qualification, and years of experience) on dependent variables (domains of teledentistry). A *p* value of ≤0.05 was taken as statistically significant.

## 3. Results

This observational study consisted of 506 participants. The response rate of participation was 89.39%. There was 266 (52.56%) female and 240 (47.43%) males in this study. The age range of participants was from 20 to 64 years. There were 340 (67.2%) participants from 20- to 34-year age bracket, 133 (26.3%) belonged to 35 to 44 years, 13 (2.6%) were from 45- to 54-year age group, and 20 (4.0%) were from 55- to 64-year age bracket. Qualification-wise, the majority of the 259 (51.2%) participants were general dental practitioner, and 172 (34.0%) participants were consultant/specialist. Regarding the experience, majority of the 306 (60.5%) participants had 1–5-year experience, 104 (20.6%) participants had 6–10 years' experience, and 55 (10.9%) participants had 11–15 years of experience.

**Figure 1:**
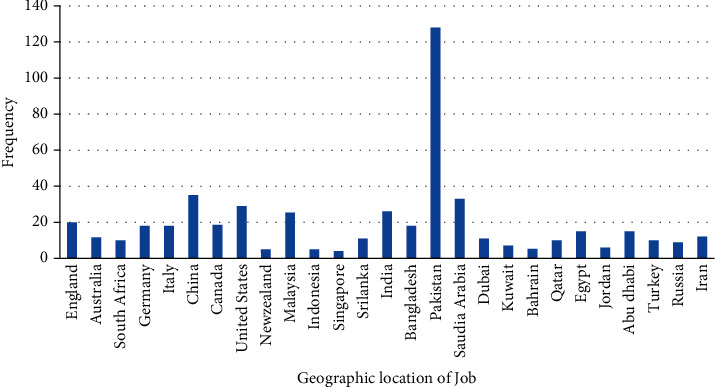
Distribution of responses from different countries.

In this study, the majority of the responses were recorded from South Asia 183 (36.16%), 65 (12.84%) from continental Europe, 87 (17.19%) from Western Asia, 34 (6.71%) from East Asia, and 29 (5.73%) from the United States of America as shown in Figure 1.

Furthermore, most of the dentists 247 (48.81%) worked in a private setup, while 122 (24.11%) worked in a public sector and the remaining 137 (27.07%) were working in academic institutes. In this study, majority of the respondents 191 (37.7%) worked 35–49 hours per week, whereas 170 (33.6%) worked 1–19 hours per week, and 118 (23.3%) worked 20–34 hours. Regarding the daily use of the internet in clinical practice, the majority 249 (49.2%) selected 2–4 hours, while 168 (33.2%) participants were using it for less than 1 hour.

Table 1 presents the concern of participants about data security and patient consent. In this regard, the majority of the 410 (81.02%) participants were concerned about gaining patient consent, whereas 54 (10.7%) were not feeling either way. However, most of the 471 (93.08%) respondents were concerned about the confidentiality of online data sent by patients but 28 (5.53%) were not concerned. When asked about digital forgery, more than three-fourths (79.64%) of the participants were concerned about it and 35 (6.91%) were not concerned about digital forgery. Furthermore, the majority of the 436 (86.16%) participants were concerned about hardware and software incompatibility in teledentistry, although 28 (5.53%) were not concerned about it. Regarding the reliability of teledental equipment, many of the 436 (86.16%) participants were concerned about it; however, a small number of 14 (2.76%) participants were not concerned.

Table 2 shows the responses on impact of teledentistry to improve dental practice. In this regard, the majority of the 256 (50.6%) participants were not in favor of teledentistry used for clinical diagnosis, whereas about one-fourth (25.29%) of the participants disagreed. Furthermore, more than three-fourths (79.64%) of the participants agreed that teledentistry would help shorten their clinic waiting list, whereas a small number of 47 (9.28%) respondents disagreed. Regarding the question about teledentistry capability to enhance dental guidelines and advice, the majority of the 342 (67.58%) participants were in favor of it, but less than one-fourth (22.9%) were not feeling either way. However, most of the 326 (64.42%) participants agreed that teledentistry will improve the interaction between peers, although 55 (10.86%) disagreed on it. Concerning that teledentistry would provide a safe atmosphere for practicing dentistry (e.g., COVID-19 pandemic), majority of the 416 (82.21%) participants agreed on it but a small number of the 28 (5.53%) participants disagreed. Additionally, more than two-thirds (71.73%) of the participants agreed that teledentistry would make patient's referral more efficient, although 61 (12.05%) disagreed on this.

Table 3 describes the application and usefulness of teledentistry in dental practice. Out of 506 participants, more than half of the participants (51.18%) agreed that teledentistry would enhance clinical training and continuing dental education, while 82 (16.2%) disagreed, and 165 (32.6%) were neutral about that. Regarding cost-effectiveness, the majority of the 354 (69.96%) participants agreed that the teledentistry would reduce costs for the dental practices, while 35 (6.9%) disagreed on it. Concerning teledentistry, it would increase treatment time spent with the patient; most of the 272 (53.75%) participants agreed on it. However, less than one-fourth (17.58%) disagreed. With reference to the question of teledentistry that would necessitate an extra appointment for taking photographs, the majority of the 300 (59.28%) contestants agreed, whereas 96 (18.97%) disagreed. Regarding the inquiry that teledentistry would save time compared with a referral letter, the majority of the 355 (70.15%) participants agreed on it, but a small number of 35 (6.91%) have disagreed. The concern on setup and backup of teledentistry by participants depicted that about 124 (24.50%) dentists believed that it would be an expensive option; however, the majority of the 200 (39.52%) disagreed. Lastly, whether teledentistry would be an adequate diagnostic tool in clinical practice, less than half (40.11%) of the participants agreed, and surprisingly, 193 (38.1%) were neutral.

Table 4 illustrates the application and usefulness of teledentistry for patients. The majority of the 300 (59.28%) participants agreed that teledentistry would save money for patients, while 68 (13.43%) disagreed. Furthermore, most of the 320 (63.24%) participants agreed that teledentistry would improve communication with patients, and 62 (12.25%) have disagreed. However, more than two-thirds (76.67%) of the participants agreed that teledentistry would be helpful for patient education; however, 49 (9.68%) participants disagreed. Moreover, the majority of the 423 (83.59%) participants agreed that teledentistry would help to avoid unnecessary travel to dental clinic, but a small number of 28 (5.53%) participants disagreed. More than two-thirds (69.16%) of the participants agreed that teledentistry would be helpful in monitoring the patient's condition, whereas 61 participants (12.05%) disagreed. Additionally, 244 (48.22%) participants agreed that teledentistry would be convenient and well received by patients, 68 (34.43%) disagreed, and 194 (38.3%) were neutral. Nevertheless, the majority of the 342 (67.58%) participants agreed that teledentistry would be useful for patients in remote areas, with 35 (6.91%) participants who disagreed with it. Lastly, more than half of the participants (51.18%) agreed that teledentistry should be covered by dental insurance plans, but few of the 48 (9.48%) participants have disagreed.

Table 5 presents the ANOVA test analysis. The analysis showed the statistical significance of the study participants' age, gender, qualification, and work experience with domains of teledentistry. The dentists that belonged to various age groups had a difference in opinion regarding patient's security and consent (ANOVA test; *p* value = 0.001), impact teledentistry on dental practices (ANOVA test; *p* value = 0.001), usefulness of teledentistry for patients (ANOVA test; *p* value = 0.015), and efficiency of teledentistry in dental clinics (ANOVA test; *p* value = 0.035). Similarly for qualification, a significant difference was found with all four domains of teledentistry studied (ANOVA test; *p* = 0.003, *p* = 0.001, *p* = 0.004, and *p* = 0.001), respectively. The consultants, general dentists, and resident dentists scored lower in data security and patient consent than other domains. As for work experience in years, data security and patient consent, teledentistry impact to improve dental practice and application, and usefulness of teledentistry for patients were statistically significant (ANOVA test; *p* = 0.002, 0.027, and 0.006). This could be explained by observing the mean scores between the groups, where all the experience groups scored less in data security and patient consent domain of teledentistry. On the other hand, there was no statistical significance (ANOVA test; *p* > 0.05) between gender and all domains of teledentistry.

**Figure 2:**
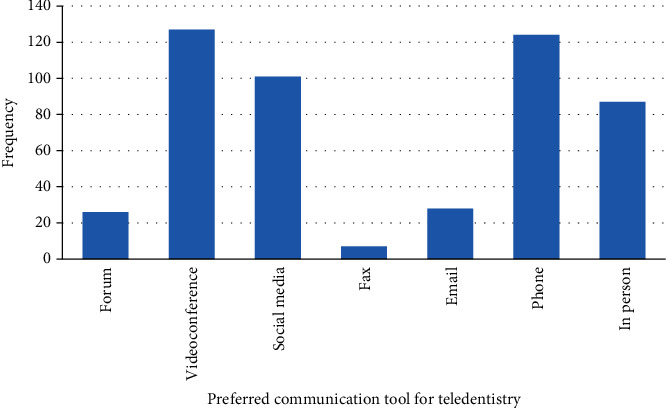
Distribution of preferred teledentistry communication tool among participants (*n* = 506).

Figure 2 shows the preferred communication tool for teledentistry. The most preferred methods of communication were videoconference 127 (25.09%), phone 124 (24.50%), social media 101 (19.96%) (WhatsApp®, Facebook®, Instagram®, skype®, Imo messenger®, snapchat®, and LinkedIn®), and in person or face to face 87 (17.19%).

**Figure 3:**
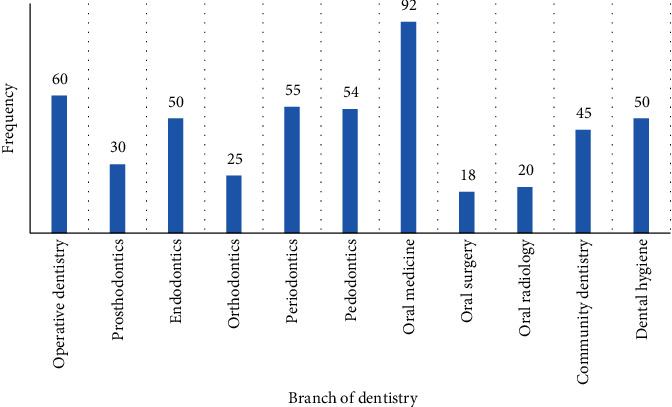
Distribution of teledentistry use in different dental specialties.

Figure 3 demonstrates the use of teledentistry in respect to different dental specialties. The majority of participants 92 (18.18%) recommended the use of teledentistry in the specialty of oral medicine. Teledentistry use was recommended in operative dentistry, the second highest by the participants 60 (11.85%). In periodontics, it was suggested by 55 (10.86%), whereas in pedodontics, 54 (10.67%) participants opted for the use of teledentistry.

## 4. Discussion

Due to the current ongoing SARS-CoV-2 pandemic, teledentistry is becoming an increasing option which is proving to be beneficial to both the patients and dentists, for their own protection. Teledentistry is capable of improving patient's access to oral health and improves delivery of oral health care and perhaps at lower costs as well [13]. Furthermore, teledentistry can also act to bridge the gap between urban and rural healthcare as well.

In this study, the majority of the participants were concerned about patients' personal data being shared over the internet, as their patients were found more comfortable sharing their data in person with dentists. These results correspond with a study with similar results about the concerns for data privacy [14]. For professionals, there is a lot of difference between teledentistry and traditional face-to-face appointments. So, the majority of the participants expressed their concern regarding their clinical diagnosis given by them using the teledentistry platform. However, these results contrast with a study where the majority (80%) of the consultants gave accurate diagnoses using teledentistry [15].

Due to the mode of transmission of COVID-19, a large number of dentists agreed that teledentistry is a safe environment to perform dentistry. These results were coinciding with study literature where dentists reported their readiness in performing teledentistry [12]. Regarding continuing clinical practice and dental education, the majority of the dentists agreed that teledentistry is useful [16]. Moreover, the majority of the dentists agreed that teledentistry is a cost-effective method for consultations, which corresponds to a study by Estai et al. [17]. This is primarily due to a smaller number of resources being used to perform teledentistry as compared to traditional appointments.

Since there are different equipment required for teledentistry, it was found in our study that appointment timings, as well as extra appointments, might be needed for patients. However, these results contrast in literature where it was found that teledentistry reduces the number of face-to-face appointments with the patients [18]. Concerning the different equipment required for teledentistry, many agreed to it being a less expensive tool. This could be due to the fact that almost all of the people are equipped with mobile phones and internet connection that is required for teledentistry [19]. Keeping in mind the current pandemic situation, the majority of the participants agreed that teledentistry is a better option than visiting a dental clinic and monitoring the patient's condition as well. This might be due to the anxiety related to contracting coronavirus when visiting the clinics [20].

Teledentistry can be particularly useful in remote areas which might not be accessible due to the lockdown situation imposed by governments worldwide. Furthermore, educating patients about their treatment and diagnosis is a vital part of their appointments with doctors. The majority of the participants agreed that teledentistry is a useful tool for patient education. These findings correlate with studies in literature where teledentistry can be beneficial for not only patient education but for dentists and dental students as well [21]. Teledentistry can be used by different dental specialties according to their use. In our study, we found that oral medicine, followed by operative dentistry, and periodontics had its most use of teledentistry. This could be due to a greater number of patients presenting with dental problems that require these specialists.

Teledentistry can be performed using many platforms such as mobile phones, video conferencing, and social media. In our study, we found that video conferencing was the most preferred method followed by phone, although few participants stated that face-to-face appointments are a better option. This could be due to a lack of awareness of the use of technology and the unavailability of tools required for teledentistry [22].

Despite the strengths of this study such as the inclusion of dentists globally, it has some limitations. Firstly, the self-administered questionnaires are prone to self-reported biasness. Lastly, the dentists working in the rural areas can be considered which could provide a better view of teledentistry in such localities.

To help manage the patients in a better way, teledentistry is becoming an emerging way for dentists to treat their patients keeping in mind the current COVID-19 pandemic. Dentists, as well as healthcare professionals, should be taught how to use teledentistry by conducting programs such as continuing dental education and awareness programs to benefit both the dentists and the patients.

## 5. Conclusion

The present study described that the dental professionals participated in this study have adequate insight and a positive attitude towards the application of teledentistry. Hence, dental professionals can be engaged in the teledentistry approach. However, the study participants showed technical perception, ethical consideration, and patient security concerns towards teledentistry. Although teledentistry is an area of expansion, there are still some barriers to its use. In particular, further research is required on the optimum modalities and the costs and benefits. With this study's limitation, further investigation is needed to understand the implementation and challenges of dental institutes and practitioners.

## Figures and Tables

**Table 1:** Distribution of responses concerning about data security and patient consent among participants (*n* = 506).

S.no	Item	Very concerned *n*%	Little concerned *n*%	Not feeling either way *n*%	Not particularly concerned *n*%	Not concerned at all *n*%
1.	Gaining patient consent for teleconsultation	295 (58.3)	115 (22.7)	54 (10.7)	21 (4.2)	21 (4.2)
2.	Confidentiality when data are sent online	337 (66.6)	134 (26.5)	7 (1.4)	7 (1.4)	21 (4.2)
3.	Potential for digital forgery	303 (59.9)	100 (19.8)	68 (13.4)	21 (4.2)	14 (2.8)
4.	Incompatible hardware and software	272 (53.8)	164 (32.4)	42 (8.3)	7 (1.4)	21 (4.2)
5.	Reliability of teledental equipment	264 (52.2)	172 (34.0)	56 (11.1)	7 (1.4)	7 (1.4)

**Table 2:** Distribution of responses about the impact of teledentistry to improve practice (*n* = 506).

S. no	Item	Disagree strongly *n*%	Disagree *n*%	Neutral *n*%	Agree *n*%	Agree strongly *n*%
1	Teledentistry would help in patient diagnosis	33 (6.5)	95 (18.8)	256 (50.6)	102 (20.2)	20 (4.0)
2	Teledentistry would help shorten the waiting list	7 (1.4)	40 (7.9)	56 (11.1)	363 (71.7)	40 (7.9)
3	Teledentistry would enhance dental guidelines and advice	7 (1.4)	41 (8.1)	116 (22.9)	295 (58.3)	47 (9.3)
4	Teledentistry would improve the interaction between peers	7 (1.4)	48 (9.5)	125 (24.7)	265 (52.4)	61 (12.1)
5	Teledentistry would provide a safe atmosphere for practicing dentistry (e.g., COVID-19 pandemic)	7 (1.4)	21 (4.2)	62 (12.3)	278 (54.9)	138 (27.3)
6	Teledentistry would make patient's referral more efficient	7 (1.4)	54 (10.7)	82 (16.2)	274 (54.2)	89 (17.6)

**Table 3:** Application and usefulness of teledentistry for dental practice (*n* = 506).

S. no	Item	Disagree strongly *n*%	Disagree *n*%	Neutral *n*%	Agree *n*%	Agree strongly *n*%
1	Teledentistry would enhance clinical training and continuing education	7 (1.4)	75 (14.8)	165 (32.6)	210 (41.5)	49 (9.7)
2	Teledentistry would reduce costs for the dental practices	7 (1.4)	28 (5.5)	117 (23.1)	284 (56.1)	70 (13.8)
3	Teledentistry would increase treatment time spent with the patient	7 (1.4)	82 (16.2)	145 (28.7)	230 (45.5)	42 (8.3)
4	Teledentistry would necessitate an extra appointment for taking photographs	14 (2.8)	82 (16.2)	110 (21.7)	273 (54.0)	27 (5.3)
5	Teledentistry would save time compared with a referral letter	7 (1.4)	28 (5.5)	116 (22.9)	307 (60.7)	48 (9.5)
6	Teledentistry would be too expensive to set up	14 (2.8)	186 (36.8)	182 (36.0)	82 (16.2)	42 (8.3)
7	Teledentistry would provide sufficient information about patient illness	28 (5.5)	82 (16.2)	193 (38.1)	163 (32.2)	40 (7.9)

**Table 4:** Application and usefulness of teledentistry for patients (*n* = 506).

S. no	Item	Disagree strongly *n*%	Disagree *n*%	Neutral *n*%	Agree *n*%	Agree strongly *n*%
1	Teledentistry would save money for patients	14 (2.8)	54 (10.7)	138 (27.3)	279 (55.1)	21 (4.2)
2	Teledentistry would improve communication with patients	32 (6.32)	30 (5.92)	124 (24.5)	272 (53.8)	48 (9.5)
3	Teledentistry would be helpful patient education	24 (4.74)	25 (4.94)	69 (13.6)	320 (63.2)	68 (13.4)
4	Teledentistry would help to avoid unnecessary travel to dental clinic	8 (1.58)	20 (3.95)	55 (10.9)	320 (63.2)	103 (20.4)
5	Teledentistry would be helpful in monitoring the patient's condition	7 (1.4)	54 (10.7)	95 (18.8)	322 (63.6)	28 (5.5)
6	Teledentistry would be convenient and well received by patients	34 (6.71)	34 (6.71)	194 (38.3)	188 (37.2)	56 (11.1)
7	Teledentistry would be useful for patients in remote areas	16 (3.16)	19 (3.75)	129 (25.5)	191 (37.7)	151 (29.8)
8	Teledentistry should be covered by dental insurance plans	24 (4.74)	24 (4.74)	199 (39.3)	204 (40.3)	55 (10.9)

**Table 5:** Comparison of independent variables with domains of teledentistry among participants (*n* = 506).

Variable	Data security and patient consent Mean (SD)	Capability of teledentistry to improve dental practice Mean (SD)	Usefulness of teledentistry for dental practice Mean (SD)	Usefulness of teledentistry for patients Mean (SD)
Age (years)				
20–34	7.81 ± 3.91	21.36 ± 4.63	23.87 ± 5.96	28.64 ± 6.64
35–44	9.66 ± 6.28	22.19 ± 5.44	23.44 ± 6.53	30.48 ± 6.29
45–54	9.82 ± 4.64	24.6 ± 5.14	24.76 ± 3.07	28.76 ± 3.07
55–64	5.65 ± 0.48	24.8 ± 3.86	25.4 ± 3.87	32.6 ± 1.92
*p* value	0.001^∗∗^	0.001^∗∗^	0.035^∗^	0.015^∗^
Gender				
Female	7.64 ± 3.68	21.84 ± 4.85	23.99 ± 6.21	29.26 ± 6.69
Male	9.9 ± 6.57	21.67 ± 5.42	23.47 ± 6.14	29.55 ± 5.88
*p* value	0.103	0.771	0.208	0.181
Qualification				
Consultant/specialist	7.8 ± 4.17	23.09 ± 5.04	24.76 ± 6.42	30.41 ± 6.06
General dental practitioner	8.43 ± 5.06	21.15 ± 4.6	23.52 ± 5.96	29.14 ± 6.49
Resident/graduate research	8.93 ± 4.98	21.04 ± 5.25	22.54 ± 5.82	27.54 ± 6.22
Other	7.99 ± 2.05	20.99 ± 1.02	24.13 ± 2.05	27.58 ± 3.59
*p* value	0.003^∗∗^	0.001^∗∗^	0.004^∗∗^	0.001^∗∗^
Work experience (in years)				
1–5	7.56 ± 3.73	21.34 ± 4.54	23.88 ± 6	28.9 ± 6.57
6–10	10.01 ± 6.19	22.07 ± 5.9	23.67 ± 5.84	29.35 ± 6.84
11–15	9.18 ± 5.78	23.48 ± 3.85	24.03 ± 4.52	30.81 ± 5.73
More than 16	7.87 ± 4.15	22.14 ± 6.13	24.78 ± 6.51	30.37 ± 3.93
*p* value	0.002^∗∗^	0.027^∗^	0.087	0.006^∗∗^

^∗^ *p* value ≤ 0.05; ^∗∗^*p* value < 0.000; SD: standard deviation.

## Data Availability

The raw data used to support the findings of this study are included within the article.
